# GOALS for Adherence with Low-cost Incentives (GOALS): Findings From Year 1 of a Randomized Controlled Trial Evaluating the Impact of Small Airtime Incentives on ART Adherence Among Young People Living With HIV in Kampala, Uganda

**DOI:** 10.21203/rs.3.rs-10776574/v1

**Published:** 2026-10-14

**Authors:** Sebastian Linnemayr, Haijing Crystal Huang, Faith Kemunto Onkundi, Barbara Mukasa, Mary Odiit

## Abstract

Background. Low adherence to antiretroviral therapy among HIV-positive youth threatens treatment outcomes, particularly in sub-Saharan Africa. Traditional incentives typically target uniformly high adherence levels and may demotivate individuals with initially low adherence. This study aims to compare subgoal incentives with individual-specific interim targets versus fixed-goal incentives with a uniform high target for improving antiretroviral therapy adherence among youth living with HIV. Methods. This parallel-group randomized controlled trial enrolled 630 participants aged 15 to 30 at Mildmay Hospital, Kampala, Uganda, between July 2022 and February 2024. Participants were randomized 1:1:1:1 to assigned subgoals, participatory subgoals, fixed goal, or usual care. Treatment arms received small airtime incentives for reaching quarterly adherence goals tracked via electronic pill boxes. Results. Mean control-group adherence was 65.3%. Compared to control, mean adherence increased by 5.9 percentage points for participatory subgoals (p = 0.04) and 6.0 for fixed goals (p = 0.03). The fixed-goal group showed a 7 percentage point increase in achieving 90% or greater adherence (p = 0.04). Subgoal groups were more likely to reach interim targets but did not show superior attainment of the ultimate adherence goal. Conclusions. Small airtime incentives improved ART adherence, but subgoaling did not outperform fixed goals in ultimate target attainment. Small airtime incentives are a low-cost way to improve ART adherence among youth living with HIV, and programs can adopt a simple fixed high-adherence target as the default. Name of trial registry: ClinicalTrials.gov Trial registration number: NCT05378607 Date of registration: May 18, 2022, retrospectively registered URL of trial registry record: https://clinicaltrials.gov/ct2/show/NCT05378607

